# Validation of the German Child Eating Behaviour Questionnaire (CEBQ) in children and adolescents with eating disorders and ADHD

**DOI:** 10.1186/s40337-026-01589-8

**Published:** 2026-05-08

**Authors:** Julia Ulbrich, Anja Hilbert, Simone Munsch, Ricarda Schmidt

**Affiliations:** 1 Department of Psychosomatic Medicine and Psychotherapy, Behavioral Medicine Research Unit, Integrated Research and Treatment Center AdiposityDiseases, Leipzig University Medical Center, Philipp-Rosenthal- Strasse 55, 04103 Leipzig, Germany; 2 German Center for Child and Adolescent Health (DZKJ), partner site Leipzig/Dresden, Leipzig, Germany; 3 Department of Psychology, Clinical Psychology and Psychotherapy, University of Fribourg, Fribourg, Switzerland; 4 Food Research and Innovation Center, Cluster Food and Mental Health/Psychology, University of Fribourg, Fribourg, Switzerland

**Keywords:** Child eating behavior questionnaire (CEBQ), Validation, Eating disorders, ADHD, Psychometric properties, Factor analysis, Assessment

## Abstract

**Background:**

The Child Eating Behaviour Questionnaire (CEBQ) is an internationally applied, parent-report questionnaire on children’s eating behaviors, but has mainly been validated in population-based samples or children with obesity. This study presents a first comprehensive validation of the German version of the CEBQ in a treatment-seeking and community-based sample including anorexia nervosa (AN), avoidant-restrictive food intake disorder (ARFID), loss of control (LOC) eating, attention-deficit/hyperactivity disorder (ADHD), and a healthy control group.

**Methods:**

The German version of the CEBQ was completed by 226 parents of children and adolescents (9 months to 17 years) in Germany and Switzerland. Factorial, convergent, and discriminant validity, internal consistency, as well as sociodemographic correlates were assessed, using objectively measured anthropometrics and well-established clinical interviews and questionnaires on eating disorders and associated psychopathology in parent- and self-report.

**Results:**

The original 8-factor structure showed acceptable model fit and acceptable to excellent internal consistency. Convergent validity was mostly supported by weight status and interview- and questionnaire-measured eating behaviors. The CEBQ subscales differentiated between groups associated with overeating (ADHD, LOC eating) versus restrictive eating (ARFID and AN).

**Conclusion:**

The results support the German version of the CEBQ as a valid and reliable tool for assessing eating behavior in youth with eating disorders and ADHD. Future research using larger and age-specific samples should examine psychometric comparability across developmental stages and may provide norms for the CEBQ to enhance its utility as a screening tool.

**Supplementary Information:**

The online version contains supplementary material available at 10.1186/s40337-026-01589-8.

## Abstract (plain language summary)

The Child Eating Behaviour Questionnaire (CEBQ) is an internationally used questionnaire completed by parents to describe different eating behaviors in children. This study tested whether the German version of the questionnaire is suitable not only for healthy children or children with obesity, as shown previously, but also for children and adolescents with eating disorders or eating difficulties, such as anorexia nervosa (AN), avoidant-restrictive food intake disorder (ARFID), loss of control (LOC) eating, and attention-deficit/hyperactivity disorder (ADHD). A total of 226 parents of children and adolescents in Germany and Switzerland completed the questionnaire. The results, as expected, showed that the questionnaire measured eight aspects of eating behavior in a reliable and consistent way. The parents’ answers corresponded well with the children’s weight and information from clinical interviews and conceptually related questionnaires. The CEBQ also revealed differences between groups of children or adolescents who tend to overeat (ADHD, LOC eating) compared to those who restrict their eating (ARFID and AN). Overall, the findings provide support for the German CEBQ as a useful tool for assessing eating behaviors in youth across clinical groups, including eating disorders and ADHD.

**Supplementary Information:**

The online version contains supplementary material available at 10.1186/s40337-026-01589-8.

## Background

Eating disorders (EDs) are characterized by significant and persistent disturbances in eating behavior associated with adverse effects on physical or psychosocial health [1]. A distinction can be made between restrictive EDs, such as anorexia nervosa (AN) and avoidant/restrictive food intake disorder (ARFID), and EDs with binge eating, such as bulimia nervosa (BN) and binge-eating disorder (BED) [1]. AN and ARFID are characterized by restrictive food intake, motivated by weight and shape concerns in patients with AN and sensory sensitivities, fear of aversive consequences, or lack of interest in eating in those with ARFID [1]. In contrast, following the 5th Edition of the Diagnostic and Statistical Manual of Mental Disorders (DSM-5), BED and BN are characterized by objective binge-eating episodes (OBEs), defined by eating objectively large amounts of food accompanied by a feeling of loss of control (LOC) over eating, either with (for BN) or without (for BED) regular compensatory behaviors [1]. According to the 11th revision of the International Classification of Diseases (ICD-11), BED may additionally include binge-eating episodes over eating a subjectively large amount of food [2], which can be referred to as subjective binge-eating episodes (SBEs). LOC eating describes the subjective perception of being unable to control eating, irrespective of the amount consumed, thus includes both OBEs and SBEs, which is especially relevant in children and adolescents [1, 3]. Additionally, other mental disorders may be associated with an increased risk of disordered eating behaviors or comorbid EDs. Specifically, children with attention-deficit/hyperactivity disorder (ADHD) may show increased risk for overeating behavior, BED, and BN [4, 5]. Reliable and valid measures to assess eating behavior across these conditions, both in self-report and parent-report, are crucial [6].

The Child Eating Behaviour Questionnaire (CEBQ), developed by Wardle et al. [7], is a well-established parent-reported questionnaire assessing the eating behavior of children in eight dimensions. Based on 35 items, four subscales depict food avoidance behaviors (Satiety Responsiveness [SR], Slowness in Eating [SE], Food Fussiness [FF], Emotional Undereating [EUE]) and four subscales cover food approach behaviors (Food Responsiveness [FR], Enjoyment of Food [EF], Desire to Drink [DD], Emotional Overeating [EOE]). The CEBQ has been translated, validated, and used in many studies worldwide (see Table 1 for an overview of CEBQ validation studies). The psychometric properties of the CEBQ have mainly been examined in community or school-based samples of children aged 1–16 years [e.g., 8, 9] and in samples enriched with treatment-seeking children with overweight or obesity aged 3–13 years [10, 11, 12]. Most validation studies focused on factorial validity, internal consistency, and associations of CEBQ subscales with child weight status. Although the CEBQ has recently been used in studies on ARFID [e.g., 13, 14] and ADHD [15, 16], it has never been validated in samples of individuals with interview-assessed EDs or ADHD. The only available study by Ko et al. [17] reported acceptable internal consistency, test-retest reliability, and sufficient item-subscale correlations for the Korean version of the CEBQ in a sample of 2–9-year-old children with parent-reported symptoms of infantile anorexia assessed via an online survey. However, factorial and convergent validity were not examined.

The factor structure of the CEBQ has not been replicated consistently. Studies using Confirmatory Factor Analysis (CFA), which is the recommended method for the factorial validation of questionnaires with a hypothesized factor structure [18], predominately showed poor to acceptable model fit for the theory-based original 8-factor structure. A modified 8-factor structure based on minor adjustments, such as cross loadings or removal of items, showed improved model fit [e.g., 8, 19]. A recent study in a large German sample of primary school children aged 6–11 years showed acceptable to good model fit for a shortened 24-item version [20]. Overall, internal consistency for the original and modified 8-factor structure was acceptable to good. Using Exploratory Factor Analysis (EFA), various factor models with 3–9 factors have been proposed, but most factor models have not been replicated [e.g., 21, 22]. However, two different 7-factor structures, combining SR and SE [e.g., 7, 10] or EOE and FR (overeating) [e.g., 23, 24] have been repeatedly replicated in previous studies and showed acceptable or good model fit (see Table 1). CFAs in samples of children aged 2–5 years in Indonesia or 5–12 years in Iceland showed a significant advantage of model fit for the 8-factor over both hypothesized 7-factor structures [9, 25].

Findings regarding age and sex differences in CEBQ subscales were not consistent, but there was a trend for boys to score higher on food approach subscales and girls to score higher on food avoidance subscales [10, 26]. Associations between children`s standardized body mass index (BMI, kg/m²) and CEBQ subscales have been demonstrated in most studies, with food approach subscales being positively and food avoidance subscales being negatively correlated with standardized BMI scores [27].

The aim of this study was to evaluate the psychometric properties of the German version of the CEBQ in children and adolescents with AN, ARFID, LOC eating, ADHD as well as a healthy control group. To our knowledge, the present study is the first to examine the psychometric properties of the CEBQ in a sample of children and adolescents with EDs and ADHD determined via clinical interview. Validating the CEBQ in children and adolescents with ADHD addresses an important gap by extending its applicability to a clinically relevant population at increased risk for dysregulated eating behaviors that may not meet full-threshold diagnostic criteria for EDs. Finally, a validation of the complete questionnaire in an exclusively German-speaking sample is lacking so far.

Specifically, this study sought to (1) examine item characteristics, (2) investigate the factor structure by comparing repeatedly proposed factor models, specifically, the original 8-factor structure [7] and two 7-factor structures that showed a combined SR and SE factor [e.g., 7, 10] or combined EOE and FR factor [e.g., 23, 24], (3) examine internal consistency, (4) evaluate the convergent validity of the CEBQ by analyzing the association between CEBQ subscales and standardized BMI as well as well-established questionnaires on eating behaviors, (5) assess the discriminant validity by analyzing differences in CEBQ subscales between children and adolescents with ARFID, AN, LOC eating, ADHD, and a healthy control group, and (6) examine differences in sex and age.

**Table 1:** Published factor structures of the CEBQ

Study	Country	Method	*N*	Age, years	Recruitment setting	Factors, model fit	Note on factors
Wardle et al., 2001 [7]	UK	PCA	208	2–9	School-based	7^a^	SE and SR combined
Viana et al., 2008 [12]	Portugal	PCA	240	3–13	School-based, healthcare setting (obesity)	6	EF and FR combined, SE and SR combined
Sleddens et al., 2008 [23]	Netherlands	PCA	135	6–7	School-based	7	EOE and FR combined
Santos et al., 2011 [10]	Chile	PCA	294	6–12	School-based, healthcare setting (obesity)	7	SE and SR combined
Svensson et al., 2011 [24]	Sweden	PCA	174	1–6	School-based	7	EOE and FR combined
Sparks and Radnitz, 2012 [22]	USA	CFA	229	2–5	School-based	7 poor-acceptable^b^	SE and SR combined
		PCA				3 (15 items)	Disinhibition, Food Interest, Undereating
Cao et al., 2012 [64]	China	PCA	219	1–1.5	Population-based	7	FR split into two factors, no EF, no SR
Loh et al., 2013 [21]	Malaysia	PCA	362	13	School-based	9	FF split into 2 factors
		CFA				8 poor-acceptable^b^	FF split into 2 factors, no SR
Mallan et al., 2013 [54]	Australia	CFA	663	1–5	School-based, population-based	8^o, m^ poor-good^b^	-
Domoff et al., 2015 [8]	USA	CFA	1002	3–4	School-based	8^o, m^ acceptable-good^b^	-
						3 poor^b^	Based on Sparks and Radnitz, 2012
Ek et al., 2016 [19]	Sweden	CFA	478	3–8	School-based, healthcare setting (obesity)	8^o^ poor^c^	-
						7 poor^c^	EOE and FR combined
						8^m^ poor-good^b^	-
Sirirassamee and Hunchangsith, 2016 [68]	Thailand	PCA	680	6–11	School-based	8	-
Njardvik et al., 2018 [9]	Iceland	Comparative CFA	560	5–12	School-based	8^o^ poor-acceptable^b^	-
						7 poor-acceptable^b^	SE and SR combined
						7 poor-acceptable^b^	EOE and FR combined
						6 poor^b^	EF and FR combined, SE and SR combined
Quah et al., 2017 [69]	Singapore	CFA	636	3	Population-based	8^o, m^ poor-acceptable^b^	-
		EFA				7	No FR
Quah et al., 2019 [70]	Singapore	CFA	1102	5–6	Population-based	8^o, m^ poor^b^	-
		EFA				6	No FR, no SR
Spahić and Pranjić, 2019 [71]	Bosnia	PCA	2500	3–10	School-based, population-based	8	-
Purwaningrum et al., 2020 [25]	Indonesia	CFA	238	2–5	Population-based	7 poor-good^b^	EOE and FR combined
						8^m^ acceptable-good^b^	-
Gebru et al., 2021 [53]	Ethiopia	CFA	542	3–6	School-based	8^m^ poor-good^b^	-
Oyama et al., 2021 [72]	Samoa	CFA	113	1 (21 months)	Healthcare setting (birth cohort)	8^o, m^ poor-acceptable^b^	-
		PCA				3 (15 items)	Dysregulated Eating, Joy of Eating, Attraction to Food
Al-Hamad et al., 2021 [73]	Saudi-Arabia	PCA	200	2–6	Healthcare setting (healthy, acute care)	8	-
Ayre et al., 2022 [74]	Vietnam	EFA	102	2–5	Healthcare setting	7	No SR
						6	EF and FR combined, no SR
Manzano et al., 2021 [75]	USA	EFA	148	8–12	Healthcare setting (overweight/ obesity)	3 (27 items)	Picky Eating, Emotional Eating, Reward-based Eating
Zhou & Sun, 2021 [76]	China	CFA	372		School-based	8^m^ poor-good	-
						8^o^ poor-acceptable	-
Hunot-Alexander et al., 2022 [77]	Mexico	CFA	842	3–13	Healthcare setting (healthy, acute care)	8^o^ poor-acceptable^b^	-
Jimeno-Martinez et al., 2022 [55]	Spain	CFA	197	3–6	School-based, healthcare setting (at risk for obesity)	8^m^ good^c^	-
Malczyk et al., 2022 [78]	Poland	PCA	151	3–10	Details not reported	8	-
Mou et al., 2024 [79]	China	PCA	11780	2–5	School-based, population-based	8	-
Peuckert et al., 2025 [80]	Brazil	EFA	205	3–13	School-based	5	-
García et al., 2025 [65]	Spain	CFA	283	6–16	School-based, population based	8^o^ poor-good^b^	-
Nigam et al., 2025 [81]	USA	EFA	814	2–12	Healthcare setting (obesity)	6	EF and FR combined, SE and SR combined
Ruden & Warschburger, 2025 [20]	Germany	EFA	1337	6–11	School-based, population-based	7 (24 items)	SE and SR combined
		EFA				8 (24 items)	-
		CFA				8 (24 items) acceptable-good^b^	-

*PCA* Principal Components Analysis, *CFA* Confirmatory Factor Analysis, *EFA* Exploratory Factor Analysis, *SR* Satiety Responsiveness, *SE* Slowness in Eating, *FF* Food Fussiness, *EUE* Emotional Undereating, *FR* Food Responsiveness, *EF* Enjoyment of Food, *DD* Desire to Drink, *EOE* Emotional Overeating

Definition of recruitment settings: School-based: Recruited through educational institutions, including preschools/ childcare centers. Population-based: Recruited to represent a defined geographic population. Healthcare setting: Recruited through hospitals, outpatient clinics, or health services. Included diagnostic groups and further specifications of sample characteristics are indicated in parentheses

^a^ result of factor analysis deviating from hypothesized factor structure based on theoretical considerations, items retained in hypothesized scales

^b^ based on following fit indices: Model-fit is acceptable if: 2.00 < χ2/df ≤ 3.00, 0.90 ≤ NFI < 0.95, 0.95 ≤ CFI < 0.97, 0.90 ≤ TLI < 0.95, 0.08 < SRMR ≤ 0.10. and 0.06 < RMSEA ≤ 0.10. Model fit is good if: χ2/df ≤ 2.00, NFI ≥ 0.95, CFI ≥ 0.97, TLI ≥ 0.95, SRMR ≤ 0.08 and RMSEA ≤ 0.06 [18, 44]

^c^ no values specified, evaluation based on own statement of the authors

^o^ original 8-factor model without modifications

^m^ modified version of the original 8-factor model, e.g., cross loadings or removal of items, use of modification indices

## Methods

### Participants and study design

Data for the present study (*N* = 230) were derived from a study on the validation of the ARFID module 2.0 for the Eating Disorder Examination (EDE) [14] and the Swiss University Study of Nutrition (SUN) [28]. The studies were approved by the local Ethics Committees (Leipzig, Germany/Fribourg, Switzerland), and informed consent and assent was provided by parents and children ≥ 8 years old.

Participants of the validation study on the ARFID module 2.0 for the EDE (*N* = 170) [14] were recruited at Leipzig University Medical Center, a support group for individuals with ARFID, advertisements on the Internet, and from the population-based Leipzig Research Center for Civilization Diseases (LIFE) Child study [29] between February 2018 and May 2021. The sample comprised a treatment-seeking group with avoidant/restrictive eating behaviors, including current and lifetime diagnoses of ARFID (*n* = 39) or AN (*n* = 24), determined using the EDE interview [30, 31], or its child version [32, 33] and the ARFID module 2.0 [14]. A control group recruited from the population following a telephone screening to exclude symptoms of EDs was matched for age and sex. Inclusion criteria involved age between 0 and 17 years and sufficient German language skills, and no specific exclusion criteria were applied.

German-speaking participants of the SUN study (*n* = 60) were recruited from primary school screenings in Switzerland between November 2011 and July 2014. The presence of LOC eating and ADHD was determined through the EDE adapted for children [32, 33] and the Schedule of Affective Disorders and Schizophrenia for School-age Children, Present and Lifetime Version [34, 35]. Inclusion criteria were an age between 8 and 13 years, regular school attendance, and sufficient German language skills. Exclusion criteria included > 1 episode of compensatory behavior during the past 3 months, AN, BN, psychotic disorders, current overweight treatment, medication affecting eating, and serious medical problems. Participants with LOC eating (*n* = 14) were required to have ≥ 3 LOC eating episodes over the past 3 months with distress and/or ≥ 2 out of 5 behavioral symptoms. Children with ADHD (*n* = 24) were required to fulfill Diagnostic and Statistical Manual of Mental Disorders, Fourth Edition, Text Revision (DSM-IV-TR) or DSM-5 criteria for ADHD, and medical treatment was allowed if intake was stable over the previous 3 months [1, 36]. Children who met the inclusion criteria for both LOC eating and ADHD were included in the LOC eating group. A control group (*n* = 22) without lifetime or current LOC eating, compensatory behavior, ED or ADHD symptoms was matched for age, sex, and BMI percentile to the LOC group.

Because a completion of at least 75% of the CEBQ items was required for inclusion in this study, *n* = 4 participants were excluded, leaving a total sample of *N* = 226 participants (see Table 2 for descriptive statistics). Child age in the total sample ranged from 9 months to 17 years, with a mean age of 9.81 (*SD* = 4.79) years. Most children had German (*n* = 163, 72.1%) or Swiss (*n* = 57, 25.2%) nationality. The mean BMI standard deviation score (BMI-SDS) was − 0.47 (*SD* = 1.31), ranging from − 4.39 ≤ BMI-SDS ≤ 3.66. Most of the children were of normal weight (*n* = 150, 66.4%).

**Table 2:** Descriptive statistics for the total sample and diagnostic subgroups

	Total sample *N* = 226 (100%)	ARFID *n* = 39 (17.3%)	AN *n* = 24 (10.6%)	LOC eating *n* = 14 (6.2%)	ADHD *n* = 24 (10.6%)	Control *n* = 124 (54.9%)
Child sociodemographics and anthropometrics						
Sex (*n*, %)						
Girls	126 (55.8%)	16 (41.0%)	23 (95.8%)	7 (50.0%)	10 (41.7%)	69 (55.6%)
Boys	100 (44.2%)	23 (59.0%)	1 (4.2%)	7 (50.0%)	14 (58.3%)	55 (44.4%)
Nationality (*n*, %)						
German	163 (72.1%)	39 (100%)	24 (100%)	0	0	99 (79.8%)
Swiss	57 (25.2%)	0	0	14 (100%)	22 (91.7%)	21 (16.9%)
Age, years (*M*,* SD*)	9.81 (4.79)	7.85 (5.24)	14.83 (1.63)	10.97 (1.02)	10.37 (1.24)	9.15 (5.03)
Age group (*n*, %)						
0–7 years	63 (27.9%)	20 (51.3%)	0	0	0	43 (34.7%)
8–13 years	113 (50.0%)	12 (30.8%)	6 (25.0%)	14 (100%)	24 (100%)	57 (46.0%)
14–17 years	50 (22.1%)	7 (17.9%)	18 (75.0%)	0	0	24 (19.4%)
Weight status (*n*, %)						
Severe underweight	34 (15.0%)	18 (46.2%)	8 (33.3%)	0	0	8 (6.5%)
Underweight	23 (10.2%)	7 (17.9%)	2 (8.3%)	1 (7.1%)	2 (8.3%)	11 (8.9%)
Normal weight	150 (66.4%)	14 (35.9%)	14 (58.3%)	8 (57.1%)	18 (75.0%)	95 (76.6%)
Overweight	10 (4.4%)	0	0	3 (21.4%)	2 (8.3%)	5 (4.0%)
Obesity	9 (4.0%)	0	0	2 (14.3%)	2 (8.3%)	5 (4.0%)
BMI, kg/m^2^ (*M*,* SD*)	17.54 (4.20)	14.8 (1.58)	17.29 (2.05)	20.17 (3.53)	18.11 (3.55)	18.03 (4.86)
BMI-SDS (*M*,* SD*)	-0.47 (1.31)	-1.54 (0.93)	-1.35 (-0.94)	0.72 (1.12)	0.12 (1.19)	-0.21 (1.20)
BMI percentile (*M*,* SD*)	39.01 (30.56)	14.02 (17.57)	18.83 (17.23)	69.32 (30.50)	52.56 (29.70)	45.34 (28.78)
Parent sociodemographics						
Age, years (*M*,* SD*)	41.13 (7.64)	37.58 (6.81)	47.71 (4.91)	43.18 (3.67)	41.07 (4.52)	40.65 (8.31)
BMI, kg/m^2^ (*M*,* SD*)	25.0 (5.69)	24.90 (6.59)	24.37 (4.51)	24.84 (5.63)	23.97 (5.14)	25.38 (5.75)
Family status (*n*, %)						
Single	42 (18.6%)	9 (23.1%)	4 (16.7%)	0	1 (4.2%)	27 (21.8%)
Married	146 (64.6%)	27 (69.2%)	11 (45.8%)	10 (71.4%)	19 (79.2%)	79 (63.7%)
Separated	9 (4.0%)	0	3 (12.5%)	0	4 (16.7%)	6 (4.8%)
Divorced	27 (11.9%)	2 (5.1%)	6 (25.0%)	4 (28.6%)	0	11 (8.9%)
Widowed	1 (0.4%)	0	0	0	0	1 (0.8%)
University degree (*n*, %)	60 (26.5%)	10 (25.6%)	7 (29.2%)	3 (21.4%)	1 (4.2%)	39 (31.5%)

German reference data [37] for the BMI-SDS were used to classify child weight status into severe underweight (BMI-SDS ≤ -1.88), underweight (-1.88 < BMI-SDS ≤ -1.28), normal weight (-1.28 < BMI-SDS < 1.28), overweight (1.28 ≤ BMI-SDS < 1.88), and obesity (BMI-SDS ≥ 1.88)

*ARFID* avoidant/restrictive food intake disorder, *AN* anorexia nervosa, *LOC* loss of control, *ADHD* attention-deficit/hyperactivity disorder, *BMI* body mass index (kg/m2), *BMI-SDS* body mass index-standard deviation score

Due to missing data, values may not sum up to *N* = 226 (100%)

A detailed age-by-diagnosis distribution in single-year increments is provided in Supplementary Table S4

### Measures

#### Child eating behaviour questionnaire

The CEBQ [7] is a 35-item parent-report questionnaire assessing child food avoidance and food approach behaviors with 8 subscales (see Background). All items are answered on a 5-point Likert Scale ranging from 1 = *never* to 5 = *always* and subscale mean scores are calculated, with higher scores indicating a stronger representation of the corresponding eating behavior. Reverse-coded items (3, 4, 10, 16, and 32) were recoded prior to all analyses by inverting the 5-point Likert scale (1 to 5, 2 to 4, 3 to 3, 4 to 2, 5 to 1). The authorized German translation of the CEBQ was performed by Anja Hilbert and controlled in a backtranslation procedure by a licensed translator, both of whom had language expertise and cultural knowledge. A congruence check confirmed consistency between the original and backtranslated versions.

#### Measures for convergent validity

##### Body mass index standard deviation score

Objectively measured body height and weight were used to calculate the BMI (kg/m^2^). Based on age- and sex-specific German normative data [37], BMI-SDS was computed. Positive correlations with food approach subscales and negative correlations with food avoidance subscales were assumed.

##### Eating disorder examination

The EDE [30, 31] and its child version [32, 33] were used to assess the number of OBEs and SBEs during the past month. Positive correlations with food approach subscales and negative correlations with food avoidance subscales were assumed.

##### EDE ARFID module 2.0

The ARFID module 2.0 for the EDE [14] in its child and parent version was used to assess the number of days with avoidant/restrictive food intake during the past month. Positive correlations with food avoidance subscales and negative correlations with food approach subscales were assumed.

##### Eating disturbances in youth-questionnaire (EDY-Q)

The EDY-Q [38, 39] is a 14-item self-report questionnaire evaluating restrictive eating disturbances on a 7-point Likert scale (0 = never to 6 = always). A total mean score can be obtained from items 1–5 and 8–12, with higher scores indicating greater restrictive eating disturbances. Cronbach`s Alpha (α) and McDonalds Omega (ω) were 0.75 and 0.73 for this sample. Positive correlations with food avoidance subscales and negative correlations with food approach subscales were assumed.

### Data analytic plan

All statistical analyses were performed using IBM SPSS Statistics version 29.0.0.0 and IBM SPSS Amos version 26 for Windows. The data analytic plan and hypotheses were pre-specified.

#### Item analyses

Item distributions were tested for normality using the Shapiro-Wilk and Kolmogorov normality tests and by item skewness and kurtosis, interpreted as normally (absolute value = 0.00), slightly non-normally (absolute value < 1.00), moderately non-normally (1.00 ≤ absolute value ≤ 2.30), and severely non-normally distributed (absolute value > 2.30) [40]. Item difficulty was calculated as *p*m = sum of item scores/ (*N* * maximal item score), with scores closer to 1 indicating easier and closer to 0 indicating more difficult items. For corrected item-subscale correlations Pearson`s *r* was assessed and interpreted as sufficient if *r* ≥ .30 [41]. Missing values of CEBQ item responses were analyzed for frequency and patterns. Little’s MCAR test was significant (*p* < .05), indicating that the data were not missing completely at random (MCAR). However, the overall proportion of missing data was very low (maximum 1.8% per item, 1–5 missing items per participant, M = 0.19, SD = 0.73; see Table 3), with no systematic pattern across items or participants and no association with observed variables, such as diagnostic group, age, or sex. Based on these findings, missingness was considered consistent with a missing at random (MAR) assumption, and missing item responses were imputed using the expectation-maximization algorithm for factor and reliability analyses.

#### Factor analyses

A CFA was conducted to compare the original 8-factor structure [7] and two previously replicated 7-factor structures that showed a combined SR and SE factor [e.g., 7, 10]) or combined EOE and FR factor [e.g., 23, 24]. Multivariate normality was assessed through Mardia’s test and rejected if the absolute value of the critical ratio (*c.r.*) was > 1.96 [42]. In this case, Bollen-Stine bootstrapping with *n* = 1000 bootstraps was applied. The factor variance was set to 1 and correlations between each of the latent variables were allowed. Initial analyses included no crossloadings, and error terms were uncorrelated.

Model fit was evaluated using various fit indices: χ2/df, Root Mean Square Error of Approximation (RMSEA), Comparative Fit Index (CFI), Normed Fit Index (NFI), Tucker-Lewis-Index (TLI), Standardized Root Mean Squared Residual (SRMR) and interpreted as acceptable if 2.00 < χ^2^/df ≤ 3.00, 0.90 ≤ NFI < 0.95, 0.95 ≤ CFI < 0.97, 0.90 ≤ TLI < 0.95, 0.08 < SRMR ≤ 0.10. and 0.06 < RMSEA ≤ 0.10 or as good if χ2/df ≤ 2.00, NFI ≥ 0.95, CFI ≥ 0.97, TLI ≥ 0.95, SRMR ≤ 0.08 and RMSEA ≤ 0.06 [18, 43, 44]. The Akaike Information Criterion (AIC) was assessed for model comparison, with lower scores indicating better model fit [45]. Χ^2^ difference tests were performed for model comparison of nested models. To identify the best-fitting model, modification indices were used to allow for crossloadings and correlated error terms if plausible in content. Factor loadings and squared multiple correlations were presented for the unmodified original 8-factor model, with factor loadings < 0.40 considered as random [46] and squared multiple correlations (variance in item responses explained by factor) interpreted as good if ≥ 0.40 [47]. Spearman rank correlation coefficients were used to compute intercorrelations between the subscales of the CEBQ, and interpreted as small (ρ ≤ 0.29), medium (0.30 ≤ ρ ≤ 0.49), and strong (ρ ≥ 0.50) [48].

All the following analyses were conducted for the unmodified original 8-factor model.

#### Reliability

Internal consistency was evaluated by Cronbach`s Alpha (α) and McDonalds Omega (ω) and considered as acceptable if α, ω ≥ 0.70, good if α, ω ≥ 0.80, and excellent if α, ω ≥ 0.90 [49].

#### Convergent validity

Spearman rank correlation coefficients were used to compute correlations between CEBQ subscales and measures for convergent validity (see 2.2.2) and interpreted according to Cohen [48]. In the case of nonsignificant Spearman correlations between CEBQ subscale mean scores and the BMI-SDS, a Multivariate Analysis of Covariance (MANCOVA) was performed on the effect of weight group (severe underweight, underweight, normal weight, overweight and obesity) on CEBQ subscale mean scores to identify non-monotonic relationships, controlling for age, sex, and diagnostic group.

#### Discriminant validity

To examine differences in CEBQ subscales among diagnostic groups (ARFID, AN, LOC eating, ADHD, control), a MANCOVA was conducted, controlled for age, sex, and BMI-SDS. In case of significant multivariate effects, the MANCOVA was followed by one-way analyses of covariance (ANCOVAs) for each CEBQ subscale and further by univariate post-hoc tests. Effect sizes (partial η²) were calculated for the ANCOVAs and interpreted as small (0.01), medium (0.06), or large (0.14) [50]. For post-hoc tests, significance was set at *p* < .05 (two-tailed) with adjustments according to Bonferroni correction, and effect sizes (Cohen`s *d*) for significant univariate associations were calculated and interpreted as small (0.2), medium (0.5), or large (0.8) [50]. For LOC eating and ADHD groups, higher mean scores were assumed in the food approach subscales and lower mean scores in the food avoidance subscales, and vice versa for ARFID and AN groups.

#### Differences in CEBQ subscale mean scores by sex and age group

Effects of sex (boys, girls) and age (0–7 years, 8–13 years, and 14–17 years) on CEBQ subscale mean scores were examined by separate MANCOVAs. The analyses were controlled for sex (except the MANCOVA on sex), age (except the MANCOVA on age), BMI-SDS, and diagnostic group. As age groups were highly confounded by diagnostic groups, only the control group (*n* = 124) was included in this analysis.

## Results

### Item analyses

Table 3 shows item characteristics and subscale mean scores of the CEBQ. Shapiro-Wilk and Kolmogorov-Smirnov tests indicated non-normality for all items (*p* < .05). Most items showed slight to moderate non-normality according to skewness and kurtosis, with exception of items 2, 13, 15 (part of the EOE subscale), and 34 (part of the FR subscale), which showed a severe positive deviation in kurtosis (-1.19 ≤ kurtosis ≤ 4.52). The item difficulty varied between 0.06 ≤ *pm* ≤ 0.60 and fell within an acceptable range, although there was a tendency towards greater difficulty. Notably, the EOE (0.06 ≤ *pm* ≤ 0.23) and the FR subscales (0.13 ≤ *pm* ≤ 0.36) contained particularly difficult items. Corrected item-subscale correlations were sufficient for all items (0.36 ≤ *r* ≤ .87).

**Table 3:** Item characteristics of the CEBQ original 8-factor model, *N* = 226

Item	Original subscale	M	SD	Skewness	Kurtosis	Item difficulty	Corrected item-subscale correlation	Missing item responses *n* (%)
1	EF	3.35	1.11	-0.40	-0.56	0.59	0.84	0 (0%)
2	EOE	1.38	0.71	1.96	3.29	0.10	0.69	3 (1.3%)
3*	SR	3.05	1.03	0.17	-0.38	0.51	0.58	2 (0.9%)
4*	SE	3.14	1.15	0.04	-0.98	0.54	0.36	4 (1.8%)
5	EF	3.47	1.09	-0.45	-0.50	0.62	0.58	0 (0%)
6	DD	2.87	1.16	0.22	-0.81	0.47	0.55	1 (0.4%)
7	FF	3.02	1.27	0.02	-1.04	0.51	0.83	1 (0.4%)
8	SE	3.06	1.23	0.20	-1.08	0.52	0.76	0 (0%)
9	EUE	2.65	1.33	0.24	-1.12	0.41	0.72	3 (1.3%)
10*	FF	3.19	1.21	-0.22	-0.80	0.55	0.83	1 (0.4%)
11	EUE	3.15	1.11	-0.22	-0.66	0.54	0.60	0 (0%)
12	FR	2.43	1.03	0.21	-0.89	0.36	0.57	0 (0%)
13	EOE	1.44	0.76	2.01	4.52	0.11	0.65	3 (1.3%)
14	FR	1.69	1.04	1.37	0.90	0.17	0.72	0 (0%)
15	EOE	1.24	0.52	2.08	3.46	0.06	0.59	1 (0.4%)
16*	FF	3.05	1.23	0.11	-0.98	0.51	0.79	0 (0%)
17	SR	2.83	1.20	0.20	-0.86	0.46	0.55	0 (0%)
18	SE	2.12	1.17	0.85	-0.21	0.28	0.61	0 (0%)
19	FR	1.61	0.93	1.57	1.92	0.15	0.72	0 (0%)
20	EF	3.24	1.07	-0.30	-0.51	0.56	0.81	0 (0%)
21	SR	3.01	0.96	-0.17	-0.56	0.50	0.67	2 (0.9%)
22	EF	3.32	1.17	-0.46	-0.58	0.58	0.85	1 (0.4%)
23	EUE	2.59	1.14	0.13	-0.85	0.40	0.53	0 (0%)
24	FF	2.53	1.12	0.42	-0.65	0.38	0.64	1 (0.4%)
25	EUE	2.57	1.29	0.21	-1.19	0.39	0.75	2 (0.9%)
26	SR	3.10	1.03	0.09	-0.57	0.53	0.68	3 (1.3%)
27	EOE	1.91	1.03	0.80	-0.49	0.23	0.55	1 (0.4%)
28	FR	2.37	1.21	0.53	-0.75	0.34	0.61	0 (0%)
29	DD	1.86	1.03	1.07	0.28	0.22	0.69	1 (0.4%)
30	SR	2.40	1.19	0.48	-0.75	0.35	0.59	3 (1.3%)
31	DD	2.31	1.22	0.55	-0.80	0.33	0.66	2 (0.9%)
32*	FF	3.38	1.16	-0.21	-0.81	0.60	0.87	1 (0.4%)
33	FF	2.86	1.27	0.10	-1.05	0.47	0.83	1 (0.4%)
34	FR	1.52	0.87	1.92	3.52	0.13	0.73	3 (1.3%)
35	SE	2.54	1.12	0.31	-0.73	0.39	0.46	2 (0.9%)

*CEBQ* Child Eating Behaviour Questionnaire, *SR* Satiety Responsiveness, *SE* Slowness in Eating, *FF* Food Fussiness, *EUE* Emotional Undereating, *FR* Food Responsiveness, *EF* Enjoyment of Food, *DD* Desire to Drink, *EOE* Emotional Overeating

* Reversed items were previously inversed

### Factor analyses

Mardia’s test indicated multivariate non-normality (*c.r*. = 20.89). Consequently, the Bollen-Stine bootstrapping method was applied. In comparison to two previously hypothesized 7-factor models, the original 8-factor model showed the best model fit, as indicated by the minimum AIC value and significant χ^2^ difference tests (*p* < .001), with fit indices indicating poor (NFI = 0.79, CFI = 0.88) to acceptable (χ^2^/df = 2.13, RMSEA = 0.07 and SRMR = 0.08) model fit (Table 4). Inspection of modification indices revealed values up to 32.87. All items showed acceptable to high factor loadings (0.43–0.94; see Table 5). The variance in item responses explained by the latent factor ranged between 18% and 88% and was interpreted as good (> 40%) for most items, except for items 4, 5, 6, 12, 17, 30, and 33.

**Table 4:** CFA of the CEBQ: Model fit for the original 8-factor model and revised factor models, *N* = 226

Model	Modifications	χ^2^/df	NFI	CFI	RMSEA	SRMR	TLI	AIC
8 factors	-	2.13 Acceptable	0.79 Poor	0.88 Poor	0.07 Acceptable	0.08 Acceptable	0.86 Poor	1329.94
8 factors	Crossloadings, correlated error terms	1.71 Good	0.83 Poor	0.92 Poor	0.06 Good	0.06 Good	0.91 Acceptable	1106.67
7 factors (SE and SR combined)	-	2.33 Acceptable	0.77 Poor	0.85 Poor	0.08 Acceptable	0.09 Acceptable	0.84 Poor	1436.36
7 factors (FR and EOE combined)	-	2.71 Acceptable	0.72 Poor	0.81 Poor	0.09 Acceptable	0.17 Poor	0.79 Poor	1650.49

*CFA* Confirmatory Factor Analysis, *CEBQ* Child Eating Behaviour Questionnaire, *CFI* Comparative Fit Index, *NFI* Normed Fit Index, *RMSEA* Root Mean Square Error of Approximation, *SRMR* Standardized Root Mean Squared Residual, *TLI* Tucker-Lewis-Index, *AIC* Akaike Information Criterion, *SR* Satiety Responsiveness, *SE* Slowness in Eating, *FR* Food Responsiveness, *EOE* Emotional Overeating

Model-fit is acceptable if: 2.00 < χ2/df ≤ 3.00, 0.90 ≤ NFI < 0.95, 0.95 ≤ CFI < 0.97, 0.90 ≤ TLI < 0.95, 0.08 < SRMR ≤ 0.10. and 0.06 < RMSEA ≤ 0.10. Model fit is good if: χ2/df ≤ 2.00, NFI ≥ 0.95, CFI ≥ 0.97, TLI ≥ 0.95, SRMR ≤ 0.08 and RMSEA ≤ 0.06 [18, 43, 44]

**Table 5:** Reliability and factorial validity of the original 8-factor model

Latent factor				Observed factor (Item)	Standardized regression weight	Variance explained by factor
Subscale	M (SD)	Cronbach`s Alpha	McDonald`s Omega			
FR	1.92 (0.81)	0.85	0.85	12	0.61	0.37
				14	0.82	0.67
				19	0.81	0.66
				28	0.65	0.43
				34	0.81	0.65
EF	3.35 (0.97)	0.89	0.90	1	0.90	0.80
				5	0.60	0.36
				20	0.87	0.76
				22	0.94	0.88
EOE	1.49 (0.61)	0.79	0.80	2	0.79	0.63
				13	0.79	0.63
				15	0.68	0.46
				27	0.68	0.47
DD	2.34 (0.96)	0.79	0.80	6	0.61	0.37
				29	0.88	0.78
				31	0.76	0.58
SR	2.86 (0.81)	0.82	0.82	3*	0.82	0.68
				17	0.56	0.32
				21	0.72	0.51
				26	0.75	0.56
				30	0.56	0.32
SE	2.71 (0.88)	0.75	0.78	4*	0.43	0.18
				8	0.90	0.80
				18	0.72	0.52
				35	0.64	0.40
EUE	2.74 (0.99)	0.82	0.83	9	0.84	0.70
				11	0.63	0.40
				23	0.55	0.30
				25	0.89	0.79
FF	3.00 (1.04)	0.91	0.92	7	0.87	0.75
				10*	0.89	0.79
				16*	0.81	0.65
				24	0.67	0.44
				32*	0.92	0.84
				33	0.84	0.71

*CEBQ* Child Eating Behaviour Questionnaire, *SR* Satiety Responsiveness, *SE* Slowness in Eating, *FF* Food Fussiness, *EUE* Emotional Undereating, *FR* Food Responsiveness, *EF* Enjoyment of Food, *DD* Desire to Drink, *EOE* Emotional Overeating

All values are significant at *p* < .001

* Reversed items were previously inversed

Based on modification indices, reasonable crossloadings and correlated error terms were allowed (see Table 6 and suppl. Table S1 for details), which further significantly (*p* < .001) improved the model fit of the 8-factor model, with some fit indices achieving a good model fit (χ^2^/df = 1.71, RMSEA = 0.06 and SRMR = 0.06). However, all items that were modified showed higher or equal factor loadings on their original subscales, while factor loadings on their new subscales were low (0.18 ≤ λ ≤ 0.44), indicating limited deviation from the hypothesized factor structure. Given the confirmatory nature of the analysis, the lack of a strong theoretical rationale for the proposed modifications, the risk of overfitting and capitalization on chance [51, 52], and to support comparability with the existing literature, the unmodified 8-factor model was retained for subsequent analyses.

**Table 6:** Crossloadings of the modified 8-factor model

Item	Original subscale (Factor loading)	New subscale (Factor loading)
24	FF (0.44)	SR (0.44)
23	EUE (0.63)	EF (0.27)
35	SE (0.52)	EUE (0.27)
5	EF (0.47)	FF (-0.26)
3*	SR (1.05)	EUE (-0.41)
33	FF (0.75)	SR (0.18)

*SR* Satiety Responsiveness, *SE* Slowness in Eating, *FF* Food Fussiness, *EUE* Emotional Undereating

* Reversed Items were previously inversed

Intercorrelations between CEBQ subscales are shown in Table 7. Significantly positive intercorrelations were found between most food approach subscales, with medium to large effect, and between food avoidance subscales, with small to large effect. Significant correlations between food approach and food avoidance subscales exhibited negative associations of up to large effect, with exception of small positive correlations between EOE and EUE and as well between DD and EUE. DD displayed nonsignificant correlations with most subscales, except for small to medium positive associations with EUE, EOE, and FR.

**Table 7:** Spearman rank correlation coefficients of CEBQ subscales for the original 8-factor model

Subscale	FR	EF	EOE	DD	SR	SE	EUE	FF
FR	1							
EF	0.54**	1						
EOE	0.57**	0.38**	1					
DD	0.34**	0.11	0.33**	1				
SR	− 0.37**	− 0.66**	− 0.25**	0.01	1			
SE	− 0.31**	− 0.42**	− 0.18**	0.08	0.50**	1		
EUE	0.03	− 0.10	0.26**	0.15*	0.32**	0.26**	1	
FF	− 0.11	− 0.56**	− 0.08	− 0.02	0.45**	0.23**	0.24**	1

*CEBQ* Child Eating Behaviour Questionnaire, *SR* Satiety Responsiveness, *SE* Slowness in Eating, *FF* Food Fussiness, *EUE* Emotional Undereating, *FR* Food Responsiveness, *EF* Enjoyment of Food, *DD* Desire to Drink, *EOE* Emotional Overeating

* *p* < .05, ** *p* < .01

### Reliability

All subscales of the CEBQ showed acceptable to excellent internal consistency, with Cronbach`s α ranging between 0.75 ≤ α ≤ 0.91 and McDonald`s ω ranging between 0.78 ≤ ω ≤ 0.92 (Table 5).

### Convergent validity

All significant correlations corresponded to the hypothesized directions (Table 8). The number of days with avoidant/ restrictive food intake as assessed by parent- or self-report significantly showed small to medium-sized correlations with food avoidance subscales, except for the SE subscale, while correlations with most of the food approach subscales were nonsignificant, except for the EF subscale showing small (for self-report) or medium-sized (for parent-report) negative correlations. OBEs were significantly positively correlated with the subscales FR and EF, and negatively correlated with the subscales SR and SE, with small effects. Correlations between SBEs and CEBQ subscales were nonsignificant. The EDY-Q total mean score significantly showed small to large correlations with CEBQ subscales, with positive associations with food avoidance and negative associations with food approach subscales, except for EUE and DD. CEBQ subscales showed significantly small to medium-sized correlations with the BMI-SDS, with positive associations for food approach and negative associations for food avoidance subscales, with exception of the DD subscale. Overall, the DD subscale showed no significant correlations with measures of convergent validity.

To further explore possible non-monotonic relationships between weight status and CEBQ subscale mean scores, a MANCOVA was conducted. Weight groups differed significantly in the CEBQ subscales, *p* < .001, *F*(32, 852) = 3.44. The higher the weight status, the higher were the mean values of the food approach subscales and the lower the mean values of the food avoidance subscales, except for the DD subscale. Specifically, significant group differences were found for all subscales except for EOE and DD, with medium to large effects (Suppl. Table S2). Post-hoc tests showed small to large effects (Suppl. Figure S1).

**Table 8:** Measures for convergent validity of the CEBQ original 8-factor model: Spearman rank correlation coefficients

Measure	*n*	FR	EF	EOE	DD	SR	SE	EUE	FF
BMI-SDS	226	0.37**	0.43**	0.21**	− 0.03	−0.48**	− 0.36**	− 0.19**	− 0.19**
Days with avoidant/restrictive food intake, parent-report	166	− 0.13	− 0.42**	− 0.06	− 0.03	0.30**	0.11	0.24**	0.43**
Days with avoidant/restrictive food intake, self-report	127	0.10	− 0.28**	0.02	0.02	0.29**	0.06	0.24*	0.40**
OBEs	158	0.27**	0.24**	0.12	0.11	−0.25**	− 0.21**	− 0.06	− 0.06
SBEs	158	0.04	0.07	0.13	0.07	0.04	0.05	0.08	0.04
EDY-Q total mean score	128	− 0.24**	− 0.60**	− 0.27**	− 0.03	0.57**	0.38**	0.10	0.48**

*BMI-SDS* body mass index standard deviation score, *CEBQ* Child Eating Behaviour Questionnaire, *EDY-Q* Eating Disturbances in Youth-Questionnaire, *SR* Satiety Responsiveness, *SE* Slowness in Eating, *FF* Food Fussiness, *EUE* Emotional Undereating, *FR* Food Responsiveness, *EF* Enjoyment of Food, *DD* Desire to Drink, *EOE* Emotional Overeating, *OBEs* Objective binge-eating episodes, *SBEs* Subjective binge-eating episodes

* *p* < .05 (two-tailed)

** *p* < .01 (two-tailed)

### Discriminant validity

The diagnostic groups differed significantly in CEBQ subscales, *F*(32, 852) = 3.09, *p* < .001. Significant group differences were seen for most subscales, except for DD and EUE (*p* > .05) (*p* < .05; see Table 9), with medium to large effects.

**Fig. 1:**
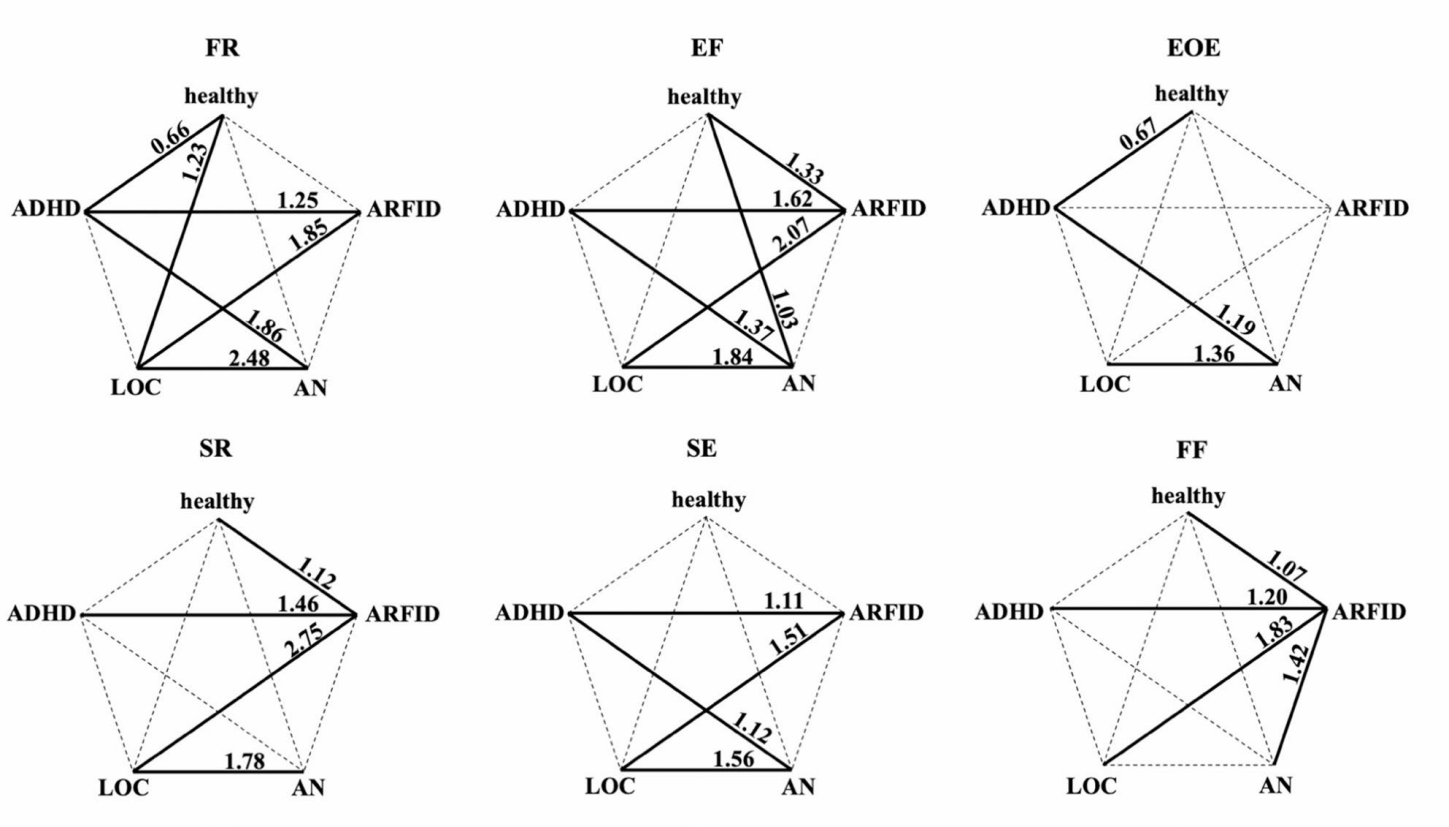
Differences in original CEBQ subscales by diagnostic groups, controlled for sex, age and BMI-SDS. *CEBQ* Child Eating Behaviour Questionnaire, *SR* Satiety Responsiveness, *SE* Slowness in Eating, *FF* Food Fussiness, *EUE* Emotional Undereating, *FR* Food Responsiveness, *EF* Enjoyment of Food, *DD* Desire to Drink, *EOE* Emotional Overeating. Continuous lines indicate significant group differences (*p* < .05, adjustments according to Bonferroni-correction). Numbers indicate the effect size (Cohen`s *d*). No significant group differences were found for DD and EUE

Post-hoc tests revealed medium to large significant univariate effects (see Fig. 1). As expected, significantly more food approach behaviors were seen for LOC and ADHD groups relative to ARFID and AN, while the latter showed greater food avoidance behaviors. Thus, significant differences emerged between groups associated with overeating versus restrictive eating patterns. In contrast, none of the subscales significantly differentiated within these subgroups, that is, between LOC and ADHD or between ARFID and AN. The only exception was the FF subscale, which significantly distinguished ARFID from all other groups, including AN, with the highest FF values observed in the ARFID group.

**Table 9:** Differences in original CEBQ subscales by diagnostic groups, controlled for sex, age, and BMI-SDS

CEBQ subscale	Control *n* = 124		ARFID *n* = 39		AN *n* = 24		LOC *n* = 14		ADHD *n* = 24			ANCOVAs		
	M	SD	M	SD	M	SD	M	SD	M	SD		F(4, 217)	*p*	η_*p*_²
FR	1.94^a^	0.76	1.61^a^	0.57	1.30^a^	0.33	2.90^b^	0.98	2.45^b^	0.81		7.63	< 0.001	0.12
EF	3.55^a^	0.79	2.44^b^	0.96	2.71^b^	0.96	4.25^a^	0.55	3.83^a^	0.65		12.75	< 0.001	0.19
EOE	1.47^a, b^	0.61	1.34^a, c^	0.38	1.21^a^	0.34	1.96^b, c^	0.80	1.89^c^	0.73		4.80	< 0.001	0.08
DD	2.27	0.87	2.52	1.11	2.21	1.18	2.43	0.88	2.51	0.93		0.57	0.69	0.01
SR	2.77^a, b^	0.76	3.59^c^	0.62	3.24^b, c^	0.81	2.00^a^	0.43	2.58^a, b^	0.80		7.64	< 0.001	0.12
SE	2.67^a, b^	0.82	3.17^a^	0.88	3.17^a^	0.89	1.95^b^	0.54	2.21^b^	0.83		3.44	< 0.001	0.09
EUE	2.61	0.97	3.28	1.01	2.48	0.96	2.54	0.72	2.90	0.94		1.65	0.16	0.03
FF	2.87^a^	1.02	3.91^b^	0.78	2.76^a^	0.86	2.49^a^	0.77	2.81^a^	1.11		6.68	< 0.001	0.11

*CEBQ* Child Eating Behaviour Questionnaire, *SR* Satiety Responsiveness, *SE* Slowness in Eating, *FF* Food Fussiness, *EUE* Emotional Undereating, *FR* Food Responsiveness, *EF* Enjoyment of Food, *DD* Desire to Drink, *EOE* Emotional Overeating

^a, b, c^ Identical letters indicate nonsignificant differences between the respective groups. *p* < .05, adjustments according to Bonferroni-correction

### Differences in CEBQ subscale mean scores by sex and age group

No significant differences were found in the CEBQ subscales by sex, *F*(16, 226) = 2.44, *p* = .18. Effect sizes for all subscales were small (η_p_² ≤ 0.01), indicating negligible differences between boys and girls. Age groups differed significantly in CEBQ subscales, *F*(16, 226) = 2.44, *p* = .002. Univariate analyses showed significant group differences for all food avoidance subscales (*p* < .05) with small to medium effect (Suppl. Table S3), but no significant differences for food approach subscales. Food avoidance scores were significantly higher in the 0–7 than 8–13 years age group for SR, SE, and EUE (medium effects), and compared to the 14–17 years age group for FF and SR (large effects).

## Discussion

This is the first study examining the validity and reliability of the CEBQ in children and adolescents with interview-assessed EDs such as AN and ARFID as well as LOC eating and ADHD. Overall, the CEBQ showed acceptable validity and reliability. Compared to two previously proposed 7-factor models [e.g., 7, 23], the original 8-factor structure [1] exhibited the best fit for this sample, with acceptable to high factor loadings across all items. Internal consistency for all subscales was acceptable to excellent. As expected, convergent validity, as assessed through objectively derived BMI-SDS and well-established questionnaires, was acceptable across all subscales except for the DD subscale. The CEBQ subscales, except for DD and EUE subscales, significantly differentiated between diagnostic groups associated with overeating (ADHD and LOC) and those characterized by restrictive eating (ARFID and AN), as hypothesized. No significant differences in CEBQ subscale mean scores were observed for child sex, whereas food avoidance behavior varied across age groups.

Missing item responses occurred rarely suggesting good comprehensibility and applicability of the CEBQ to the diverse sample including eating disturbances and ADHD. Correlations between each item and its subscale were sufficient for all items, as previously reported in a sample of Dutch primary school children [23]. Items of the subscales EOE and FR showed increased difficulty, reflected in low mean scores and positive skewness, as previously reported for the EOE subscale [e.g., 23, 53]. Particularly younger children may tend to undereat rather than overeat in emotional situations [7, 23].

Comparative factor analyses revealed the superiority of the theory-based original 8-factor model over two previously identified 7-factor structures, combining SE and SR factors [7, 10], or combining EOE and FR factors [23, 24], as previously demonstrated in nonclinical samples [9, 25]. Regarding the CEBQ’s factorial validity, previous studies in nonclinical samples showed comparable results with poor to acceptable model fit of the original 8-factor model [e.g., 9], acceptable to high factor loadings [e.g., 8, 9], and acceptable to excellent internal consistency for all subscales [e.g., 7, 9]. The similarity of the CFA results between the clinical sample of the current study and non-clinical samples suggests that the CEBQ is universally applicable to both healthy and treatment-seeking individuals. As demonstrated previously, the CEBQ’s model fit was improved using modification indices [e.g., 8, 19]. Some of the applied crossloadings in the present study (see Table 6) have already been reported in other studies, for example for Item 24 [54] and Item 5 [55]. Although the modified 8-factor model showed an improved model fit to our data with up to good fit indices, analyses for convergent and discriminant validity were conducted using the unmodified 8-factor model. Given that all modified items had equal or higher factor loadings on their original subscales and the factor loadings on the new subscales were low, the unmodified model was retained to avoid randomness and possible overfit to the data.

Aligning meta-analytic findings [27], CEBQ subscales correlated significantly positively (for food approach subscales) or negatively (for food avoidance subscales) with children’s BMI-SDS. Only the DD subscale did not correlate with children`s BMI-SDS, as previously indicated [e.g., 8, 56]. Additionally, this study used interview- and questionnaire-based measures on children’s EDs and associated psychopathology for evaluating convergent validity of the CEBQ. As expected, the self-reported number of OBEs assessed by interview was significantly positively correlated with the subscales FR and EF and negatively with the subscales SR and SE, indicating an association between binge eating and general overeating behavior in children and adolescents, consistent with laboratory-based test meal studies in children with LOC eating [e.g., 57, 58]. The correlation between the number of self-reported OBEs and the EOE subscale was nonsignificant, although negative affect has been proposed as an antecedent of overeating and LOC eating in children and adolescents [59]; however, test meal and ecological momentary assessment studies have not consistently supported this association [57, 60]. In contrast to OBEs, the number of SBEs did not correlate significantly with any of the CEBQ subscales, as the CEBQ is based on parent-report and parents might have difficulties noticing SBEs in their children.

The parent-reported number of days with avoidant-restrictive food intake showed significantly medium-sized positive and negative correlations with the SR, FF, and EF subscales, corresponding to avoidant-restrictive food intake being defined by restrictions in the quantity (measured by SR) or variety (measured by FF) of food intake [14], and reflecting clinical observations that children with avoidant-restrictive food intake hardly enjoy eating (measured by EF). Overall, all significant correlations between the CEBQ subscales and the measures for convergent validity corresponded to the hypothesized directions, which supports convergent validity. One exception is the DD subscale, which showed no significant associations with measures for convergent validity and inconsistent intercorrelations with other CEBQ subscales, in line with previous research [e.g., 23, 54].

Regarding discriminant validity, significant differences by diagnostic group were seen for all CEBQ subscales, except for the subscales DD and EUE, indicating that these latter subscales may not be suitable for discriminating different eating disturbances, ADHD, and controls. Despite adequate internal consistency and factor loadings for the DD subscale in the present sample, its weak subscale intercorrelations as well as low convergent and discriminant validity across different samples [e.g., 8, 54] suggest a psychometric limitation of the subscale.

Mean values of the food approach subscales were particularly low for AN and ARFID groups and high for LOC and ADHD groups, and for food avoidance subscales vice versa, while the control group showed medium values for all subscales. These results were expected given that restrictive eating behavior is a diagnostic criterion for ARFID and AN, while overeating is diagnostically relevant for BED [1] and common in children with ADHD [5, 61]. The results thus show that the CEBQ can distinguish between diagnostic groups associated with overeating (ADHD and LOC) and those involving restrictive eating (ARFID and AN) but reveals only limited discrimination between groups within each of these categories. Of note, the FF subscale significantly differentiated ARFID from all other diagnostic groups, including AN, which is consistent with the understanding that selective eating behavior may be a main characteristic feature of ARFID [1]. Given small subgroup sizes, particularly in the overweight, obesity, and LOC eating groups (*n* < 20), findings regarding diagnostic differentiation should be interpreted with caution.

In terms of sociodemographic correlates, the present findings align with previous studies [e.g., 24] on absent sex differences, while general evidence base is mixed [e.g., 7, 10]. Regarding children’s age, only food avoidance subscales significantly differed by age group, with highest scores for children aged 0–7 years, which is consistent with findings that the peak of picky eating and food neophobia is usually seen between ages 2–6 years [62], and decreasing food avoidance and constant food approach behavior from toddlers to young teenagers in CEBQ studies covering a wide age range [12, 63].

Regarding strengths and limitations, one of the strengths of this study is the sample of children and adolescents with interview-assessed AN, ARFID, LOC eating, ADHD, and a control group of healthy children. Based on a literature review, a comparative CFA containing the most frequently proposed factor models was carried out. Additionally to the objectively measured anthropometrics, well-established and validated questionnaires on eating behavior were used to determine the convergent validity of the CEBQ subscales. The inclusion of both treatment-seeking and non-treatment-seeking participants enhances the generalizability of findings to diverse populations but may also introduce variability that complicates the interpretation of results. The wide age range of 9 months to 17 years may also be a strength and limitation at the same time. Although there is no official age limit for the CEBQ, it was developed in a sample including children aged 2–9 years [7] and has so far mainly been validated for the age range from 12 months – 13 years [e.g., 12, 64], with exception of one recent validation study in Spanish children and adolescents aged 6–16 years [65]. Its application in infants and adolescents remained exploratory, and some items may be interpreted differently across developmental stages, potentially introducing age-related measurement bias. Another limitation of this study pertains to the sample size, particularly in relation to the limited number of participants in the diagnostic, weight, and age groups analyzed by MANCOVAs. Finally, the results of this study exhibit limited generalizability on populations from non-Western countries and populations of lower educational levels, as the parents surveyed in this study showed above-average levels of education [66, 67].

## Conclusion

In sum, the findings provide support for the German version of the CEBQ as a valid and reliable tool for assessing eating behavior in children and adolescents across various clinical groups, including specific EDs, eating disturbances and ADHD, and can thus be used in future research and clinical settings. Given the broad developmental range of the sample (0–17 years), the present psychometric findings cannot be generalized uniformly across ages and should primarily be interpreted at the overall sample level. Future research should conduct measurement invariance analyses in large clinical samples to ensure that the results are equally transferable to all age and diagnostic groups. The provision of population-based and clinical normative scores for the CEBQ mean scores is crucial for evaluating its potential utility as a screening tool for identifying atypical eating behavior indicative of overeating vs. restrictive EDs.

## Supplementary Information

Below is the link to the electronic supplementary material.

Supplementary Material 1.

## Data Availability

The data are available from the author upon reasonable request.

## Abbreviations

ADHD: Attention-deficit/hyperactivity disorder
AIC: Akaike information criterion
AN: Anorexia nervosa
ANCOVA: One-way analysis of covariance
ARFID: Avoidant-restrictive food intake disorder
BED: Binge-eating disorder
BMI: Body mass index
BMI-SDS: BMI standard deviation score
BN: Bulimia nervosa
CEBQ: Child eating behaviour questionnaire
CFA: Confirmatory factor analysis
CFI: Comparative fit index
DD: Desire to drink
DSM-IV-TR: Diagnostic and statistical manual of mental disorders, fourth edition, text revision
DSM-5: Diagnostic and statistical manual of mental disorders, 5th edition
EDE: Eating disorder examination
EDs: Eating disorders
EDY-Q: Eating disturbances in youth-questionnaire
EF: Enjoyment of food
EFA: Exploratory factor analysis
EOE: Emotional overeating
EUE: Emotional undereating
FF: Food fussiness
FR: Food responsiveness
ICD-11: International classification of diseases 11th revision
LIFE: Leipzig research center for civilization diseases
LOC: Loss of control
MANCOVA: Multivariate analysis of covariance
MCAR: Missing completely at random
NFI: Normed fit index
OBEs: Objective binge-eating episodes
RMSEA: Root mean square error of approximation
SBEs: Subjective binge-eating episodes
SE: Slowness in eating
SR: Satiety responsiveness
SRMR: Standardized root mean squared residual
SUN: Swiss university study of nutrition
TLI: Tucker-Lewis-index
