# Supplementary material for: Validation of the German Child Eating Behaviour Questionnaire (CEBQ) in children and adolescents with eating disorders and ADHD
Source: J Eat Disord. 2026 May 8;14:108. doi: 10.1186/s40337-026-01589-8 (PMC13156854; doi:10.1186/s40337-026-01589-8)
Supplement: Supplementary file 1 — Supplementary Material 1. [file 40337_2026_1589_MOESM1_ESM.pdf]

Validation of the German Child Eating Behaviour Questionnaire (CEBQ) in a diverse clinical sample including eating disorders and ADHD

Supplement

Table S1

Correlated error terms of the modified 8-factor model

Items with correlated error terms	Subscales
17 – 30	SR
6 – 12	DD, FR
27 – 13	EOE
19 – 34	FR
14 – 12	FR

Note. Abbreviations: SR, Satiety Responsiveness; FR, Food Responsiveness; DD, Desire to Drink; EOE, Emotional Overeating.

Table S2

Differences in CEBQ subscales by weight group, controlled for sex, age, and diagnostic group

CEBQ Subscale	Severe underweight $n = 34$		Underweight $n = 23$		Normal weight $n = 149$		Overweight $n = 10$		Obesity $n = 9$		ANCOVAs		
	M	*SD*	*M*	*SD*	*M*	*SD*	*M*	*SD*	*M*	*SD*	$F(4, 217)$	*p*	Partial η^2
FR	1.50[a]	0.50	1.85[a,b]	0.78	1.91[a,b]	0.70	2.52[b,c]	1.02	3.40[c]	1.28	12.58	<.001	.19
EF	2.50[a]	0.89	3.25[b]	0.95	3.47[b]	0.91	3.98[b]	0.56	4.00[b]	0.76	10.67	<.001	.16
EOE	1.32	0.33	1.49	0.49	1.50	0.63	1.78	0.88	1.83	0.90	1.57	.18	.03
DD	2.49	1.07	2.38	0.88	2.30	0.92	1.90	1.01	2.89	1.08	1.63	.17	.03
SR	3.57[a]	0.54	3.27[a,b]	0.89	2.76[b,c]	0.77	2.31[c]	0.73	2.27[c]	0.98	11.89	<.001	.18
SE	3.24[a]	0.84	3.03[a,b]	0.79	2.60[b]	0.83	2.48[a,b]	0.96	2.17[b]	1.26	5.18	<.001	.09
EUE	3.12[a]	0.91	3.02[a,b]	0.92	2.67[b]	0.98	2.33[a,b]	1.13	2.11[b]	0.88	4.35	<.01	.07
FF	3.51[a]	1.06	3.23[a,b]	0.93	2.89[b]	1.02	2.82[a,b]	0.81	2.78[a,b]	1.29	3.56	.01	.06

Note. Abbreviations: CEBQ, Child Eating Behaviour Questionnaire; SR, Satiety Responsiveness; SE, Slowness in Eating; FF, Food Fussiness; EUE, Emotional Undereating; FR, Food Responsiveness; EF, Enjoyment of Food; DD, Desire to Drink; EOE, Emotional Overeating.

[a, b, c] Identical letters indicate nonsignificant differences between the respective groups. $p < .05$, adjustments according to Bonferroni-correction.

Table S3

Differences in original CEBQ subscales for control by age group, controlled for BMI-SDS and sex (n = 124)

CEBQ Subscale	Age 0-7 $n = 43$		Age 8-13 $n = 57$		Age 14-17 $n = 24$		ANCOVAs		
	M	*SD*	*M*	*SD*	*M*	*SD*	$F(2, 119)$	*p*	Partial η^2
FR	1.96	0.70	1.99	0.82	1.77	0.68	0.72	.49	.01
EF	3.31	0.82	3.68	0.81	3.70	0.60	1.85	.16	.03
EOE	1.28[a]	0.36	1.51[a,b]	0.65	1.70[b]	0.78	2.95	.06	.05
DD	2.44	0.96	2.11	0.81	2.33	0.83	1.78	.17	.03
SR	3.13[a]	0.64	2.57[b]	0.81	2.62[b]	0.61	6.21	<.01	.09
SE	3.01[a]	0.72	2.47[b]	0.87	2.54[a,b]	0.67	4.63	.01	.07
EUE	3.00[a]	0.96	2.42[b]	0.98	2.38[a,b]	0.75	3.48	.03	.06
FF	3.15[a]	1.01	2.86[a,b]	1.03	2.39[b]	0.81	3.34	.04	.05

Note. Abbreviations: CEBQ, Child Eating Behaviour Questionnaire; SR, Satiety Responsiveness; SE, Slowness in Eating; FF, Food Fussiness; EUE, Emotional Undereating; FR, Food Responsiveness; EF, Enjoyment of Food; DD, Desire to Drink; EOE, Emotional Overeating. [a, b, c] Identical letters indicate nonsignificant differences between the respective groups. $p < .05$, adjustments according to Bonferroni-correction.

Table S4

Age distributions for the total sample and diagnostic subgroups

Age, years	Total sample N = 226 (100%)	ARFID n = 39 (17.3%)	AN n = 24 (10.6%)	LOC eating n = 14 (6.2%)	ADHD n = 24 (10.6%)	Control n = 124 (54.9%)
0	5 (2.2%)	2 (5.1%)	0	0	0	3 (2.4%)
1	15 (5.6%)	4 (10.3%)	0	0	0	11 (8.9%)
2	11 (4.9%)	3 (7.7%)	0	0	0	8 (6.5%)
3	4 (1.8%)	0	0	0	0	4 (3.2%)
4	8 (3.5%)	2 (5.1%)	0	0	0	6 (4.8%)
5	9 (4.0%)	5 (12.8%)	0	0	0	4 (3.2%)
6	7 (3.1%)	1 (2.6%)	0	0	0	6 (4.8%)
7	4 (1.8%)	3 (7.7%)	0	0	0	1 (0.8%)
8	9 (4.0%)	3 (7.7%)	0	0	3 (12.5%)	3 (2.4%)
9	13 (5.8%)	0	0	3 (21.4%)	7 (29.2%)	3 (2.4%)
10	26 (11.5%)	2 (5.1%)	0	5 (35.7%)	6 (25.0%)	13 (10.5%)
11	27 (11.9%)	4 (10.3%)	0	4 (28.6%)	5 (20.8%)	14 (11.3%)
12	19 (8.4%)	0	1 (4.2%)	2 (14.3%)	3 (12.5%)	13 (10.5%)
13	19 (8.4%)	3 (7.7%)	5 (20.8%)	0	0	11 (8.9%)
14	16 (7.1%)	2 (5.1%)	6 (25.0%)	0	0	8 (6.5%)
15	9 (4.0%)	0	3 (12.5%)	0	0	6 (4.8%)
16	14 (6.2%)	4 (10.3%)	3 (12.5%)	0	0	7 (5.6%)
17	11 (4.9%)	1 (2.6%)	6 (25.0%)	0	0	3(2.4%)

Abbreviations: ARFID, avoidant/restrictive food intake disorder; AN, anorexia nervosa; LOC, loss of control; ADHD, attention-deficit/hyperactivity disorder.
Due to missing data, values may not sum up to $N = 226$ (100%).

Figure S1

Differences in original CEBQ subscales by weight groups, controlled for sex, age and diagnostic group

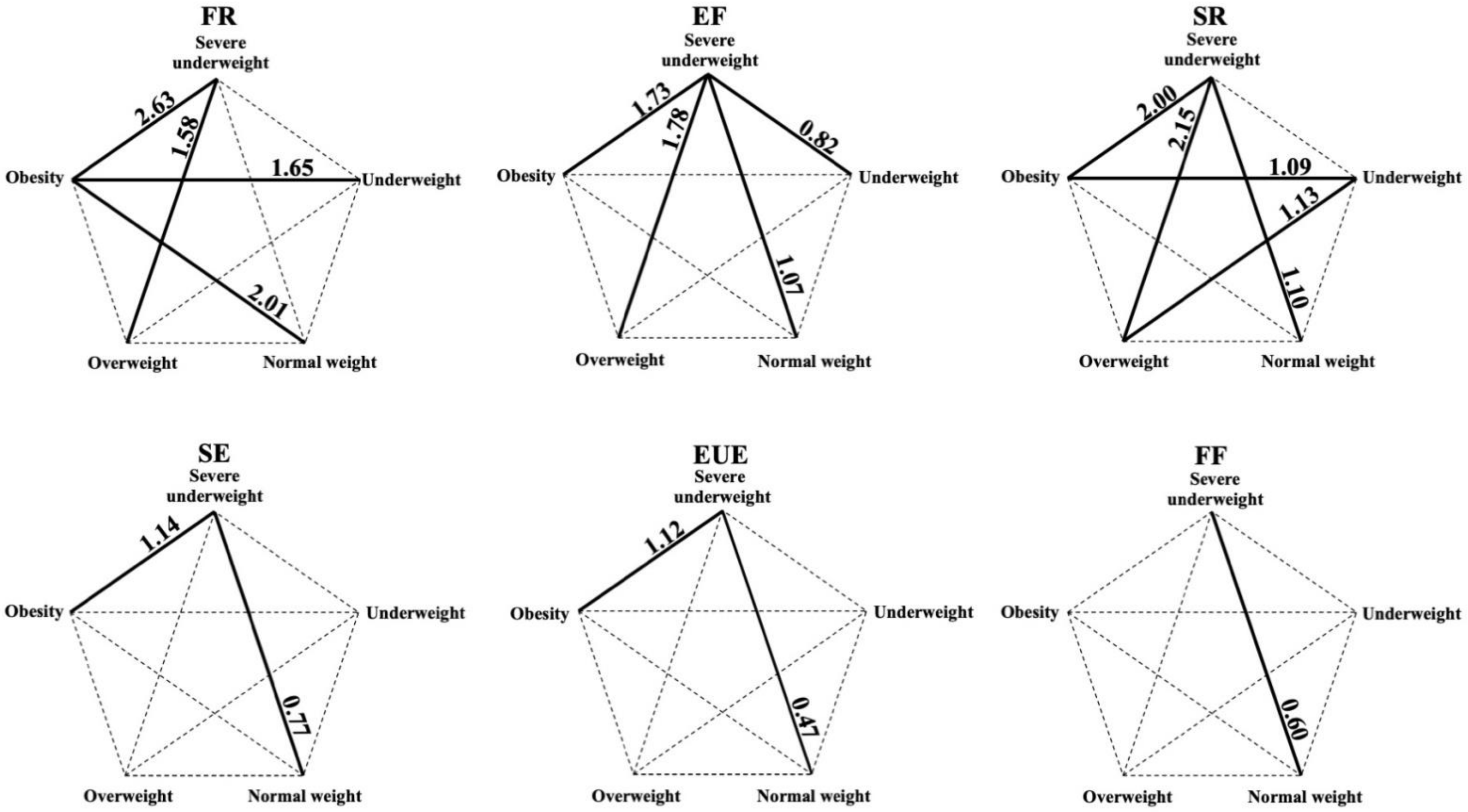

Note. Abbreviations: CEBQ, Child Eating Behaviour Questionnaire; SR, Satiety Responsiveness; SE, Slowness in Eating; FF, Food Fussiness; EUE, Emotional Undereating; FR, Food Responsiveness; EF, Enjoyment of Food; DD, Desire to Drink; EOE, Emotional Overeating. Continuous lines indicate significant group differences ($p < .05$, adjustments according to Bonferroni-correction). Numbers indicate the effect size (Cohen`s *d*). No significant group differences were found for DD and EOE.
